# The Effects of Harvest Maturity of *Eragrostis tef* ‘Moxie’ Hay and Supplemental Energy Source on Forage Utilization in Beef Heifers

**DOI:** 10.3390/ani14020254

**Published:** 2024-01-13

**Authors:** Allison V. Stevens, Cheyanne A. Myers, John B. Hall, Gwinyai E. Chibisa

**Affiliations:** Department of Animal, Veterinary and Food Sciences, University of Idaho, Moscow, ID 83844, USAcheyannem@uidaho.edu (C.A.M.); jbhall@uidaho.edu (J.B.H.)

**Keywords:** energy supplementation, forage utilization, harvest maturity, teff hay

## Abstract

**Simple Summary:**

Due to several factors including persistent drought conditions and the increased occurrence and severity of fire on rangeland, there is an increased need for the use of alternative forages to meet the nutrient requirements of ruminants raised in extensive grazing systems. However, information on the nutritive value of alternative forages like *Eragrostis teff* as influenced by harvest maturity, and the best supplementation strategies that complement and improve utilization of those forages, is still lacking. Therefore, the present study evaluated the effects of harvest maturity of teff hay (early- compared to late-heading stage of maturity) and supplemental energy sources (corn grain compared to beet pulp at 0.5% of body weight) on nutrient intake and digestibility and nitrogen utilization in beef heifers. Delaying the harvest of teff hay resulted in a decrease in nutrient supply, which was not attenuated by feeding either supplemental corn grain or beet pulp. Nitrogen retention was also negative regardless of harvest maturity, and this potentially indicates that there might be a need to provide both energy and protein supplements to improve nutrient supply and, thus, growth performance when feeding teff hay to beef cattle.

**Abstract:**

The phenological stage of maturity of grasses and supplementation program can impact forage utilization in grazing beef cattle. However, the potential interaction between harvest maturity of *Eragrostis tef* (teff) hay and energy supplement source was yet to be fully evaluated. Therefore, our objective was to determine the effects of harvest maturity of teff hay and supplemental energy sources on nutrient intake, apparent total-tract nutrient digestion, nitrogen (N) utilization, and ruminal fermentation characteristics in beef heifers. A split-plot design with teff hay harvest maturity as the whole plot and supplemental energy source as the subplot was administered in a three-period (21 d), three × three Latin square design. Six crossbred beef heifers (804 ± 53.6 kg of body weight; BW) were allocated to two harvest maturities (early- (EH]) or late-heading (LH)) and to two supplemental energy sources (no supplement (CON), or rolled corn grain or beet pulp pellet fed at 0.5% of BW). Data were analyzed using SAS. There was no harvest maturity × energy supplement interaction. Although harvest maturity had no impact on total dry matter intake (DMI), crude protein (CP) intake was greater (*p* < 0.01) for EH than LH heifers. Total intakes of dry (DM) and organic matter (OM) were also greater (*p* < 0.01) for supplemented than CON heifers, whereas acid detergent fiber (ADF) intake was greater for beet pulp heifers compared to heifers fed the CON diet and supplemental corn grain. Harvest maturity had no impact on ruminal pH. However, mean ruminal pH was lower (*p* = 0.04), duration pH < 6.2, and molar proportions of butyrate and branched-chain fatty acids were greater (*p* ≤ 0.049) for heifers fed corn grain compared to CON and beet pulp diets. Heifers fed EH hay had greater (*p* ≤ 0.02) apparent total-tract DM, OM, CP, NDF, and ADF digestibility than heifers fed LH hay. Although there was no supplemental energy effect on microbial nitrogen (N) flow, it was greater (*p* < 0.01) for EH than LH heifers. Apparent N retention, which did not differ, was negative across all diets. In summary, delaying the harvest of teff hay from the EH to LH stage of maturity compromised nutrient supply, which was not attenuated by feeding supplemental corn grain and beet pulp at 0.5% of diet DM. Because N retention was negative across harvest maturity, there might be a need to provide both energy and protein supplements to improve growth performance when feeding teff hay to beef cattle.

## 1. Introduction

The evaluation of the nutritive value of a forage, and the subsequent development of supplementation strategies that address its potential nutrient deficits, is key to optimizing production performance in beef cattle [1]. For teff grass (*Eragrostis tef*), a warm-season annual that has great appeal as an alternative forage due to its drought tolerance, fast growth rate, and high biomass yield [2], harvest maturity is a key factor that influences its nutritional quality. For instance, delaying harvest from the early-heading (EH) to the late-heading (LH) stage of maturity resulted in a decrease in crude protein (CP) content [3,4,5]. Consequently, delaying harvest has been reported to reduce nutrient supply by limiting nitrogen (N) intake, apparent total-tract N digestibility, and microbial N supply [5], thereby restricting growth performance [3]. This suggests the need for supplementation with advancing maturity to optimize production performance. However, the impact of providing supplemental protein and/or energy to beef cattle fed teff hay harvested at different stages of maturity on forage utilization and production performance remained to be determined.

In a recent study evaluating the impact of providing supplemental dried distillers grains plus solubles (DDGS; 0.5% of BW) in beef steers fed an annual or perennial forage, [6] reported a decrease in hay intake with teff (var. *Zucc*) but not Old-World Bluestem (*Bothriochloa blashii*) hay. There was also a hay type × supplement interaction for the effectively degradable fractions of organic matter (OM) and acid detergent fiber (ADF); both fractions, which were lower for steers fed teff hay, were greater for steers fed Old World Bluestem when provided supplemental DDGS. The divergent responses were attributed to differences in forage quality, with supplementation being beneficial when quality was poor (Old World Bluestem; 6.0% CP on a DM basis) but not when it was high (teff; 9.6% CP). Responses to energy supplementation could also vary depending on various factors including forage quality (high vs. low), and supplement type (starch vs. fermentable fiber) and amount [1,7]. For instance, because of its faster rate of fermentation that can result in a decrease in ruminal pH, providing a nonstructural carbohydrate (NSC; i.e., starch and sugars) compared to a fermentable fiber source could compromise fiber digestion, nutrient supply, and animal performance. However, these factors remained to be fully evaluated in beef cattle fed teff hay harvested at different stages of maturity.

Our objective was to determine the impact of the harvest maturity of teff hay (EH vs. LH) and supplemental energy source (corn grain vs. beet pulp) on nutrient intake and apparent total-tract digestibility, ruminal fermentation characteristics, and nitrogen utilization in beef heifers. We hypothesized that delaying the harvest of teff hay compromises nutrient supply and N utilization in beef cattle and this could be mitigated more effectively by feeding supplemental beet pulp than corn grain due to differences in ruminal fermentation characteristics.

## 2. Material and Methods

All animal care and handling procedures followed protocols preapproved by the University of Idaho Animal Care and Use Committee (protocol #: 2016-35) prior to initiation of study.

### 2.1. Animals and Diets

Six crossbred beef heifers (804 ± 53.6 kg) fitted with 10 cm ruminal cannulas (Bar Diamond, Inc., Parma, ID, USA) were randomly assigned to teff hay harvested at 2 stages of maturity (early- (EH) or late-heading (LH)) and different supplemental energy sources (no supplement (CON), rolled corn grain, or beet pulp pellet) (Table 1). Heifers were subjected to a split-plot, 3 × 3 Latin square design balanced for residual effects with 21 d periods (15 d adaptation and 6 d collection). The whole-plot factor was harvest maturity of teff hay, and the subplot was supplemental energy source. Agronomic practices for the teff hay fed to heifers were as described by [5]. Heifers were housed in individual tie-stalls at the University of Idaho Beef Center (Moscow, ID, USA) and were fed once daily at 0630 h to achieve ad libitum intake. Teff hay was coarsely ground through a tub grinder to ensure a uniform particle size (5 to 10 cm). A complete mineral supplement (J.R. Simplot Company, Caldwell, ID, USA) was fed daily to either meet or exceed nutrient requirements [8]. The supplement was fed free-choice and it contained 59 g/kg Ca, 60 g/kg P, 49 g/kg NaCl, 50 g/kg Mg, 3 g/kg K, 2008 mg/kg Cu, 2014 mg/kg Zn, 37.8 mg/kg Se, and 220,004 IU/kg vitamin A. Corn grain and beet pulp pellets (Midwest Agri, San Rafael, CA, USA) were provided at 0.5% of BW in a separate feeder. Throughout the study, all energy supplements that were offered daily were consumed within 1 h of feeding.

### 2.2. Measurements

Weighing of heifers was conducted before morning feeding on two consecutive days before the start and end of each experimental period. The amount of hay and supplements fed and orts remaining were recorded each day. Weekly feed samples (hay and supplements) were collected and composited by period. Each day, orts were also collected, and weekly composites stored for each animal. Prior to grinding (4 and 2 mm screen; Retsch Cutting Mill SM 200, Retsch, Haan, Germany), all samples (hay, supplement, and orts) were dried at 55 °C for 72 h.

Digesta samples were collected on d 17 (0630, 0700, 0800, 0900, 1000, 1200, 1500, 1800, and 2100 h) and 18 (0000 and 0300 h). About 1 L of digesta was collected from 4 regions of the rumen (cranial ventral, caudal ventral, central, and cranial dorsal) at each of the collection times. Samples were strained through 4 layers of cheesecloth before collection of two 5 mL aliquots. One aliquot was mixed with 1 mL of 25% metaphosphoric acid (H_2_PO_4_) for later analysis of short-chain fatty acids (SCFAs), whereas the second aliquot was mixed with 1 mL 1% H_2_SO_4_ for later analysis of ammonia-N (NH_3_-N). Indwelling pH loggers (LRCpH; DASCOR, Inc., Escondido, CA, USA) were also used to measure rumen pH every minute from d 14 to 21 of each period as described by [9].

Fecal samples were collected at 0630, 1230, and 1830 h on d 19, 0830, 1430, and 2030 h on d 20, and 1030, 1630, and 2230 h on d 21 and frozen until used to determine apparent total-tract nutrient digestibility and N balance. Corresponding spot urine samples were also collected at the same time as fecal samples. To acidify the sample and prevent loss of NH_3_-N, an 80 mL subsample of the collected urine was immediately mixed with 5 mL of 2M H_2_SO_4_. To prepare for analysis of total N, urea-N, creatinine, and purine derivatives, a 1 mL subsample of the acidified urine was diluted 1:10 with distilled H_2_O and pooled for each animal during each period before being frozen.

On the last day of each period (d 21), blood samples were collected via jugular venipuncture just prior to feeding and 3 h post-feeding. Samples were immediately centrifuged (645× *g* for 25 min at 4 °C) and plasma was harvested and frozen (−20 °C) for later analysis of urea-N (PUN).

### 2.3. Laboratory Analyses

Collected fecal samples were thawed at room temperature overnight before pooling by period for each cow. Pooled fecal samples were then dried at 55 °C for 72 h before grinding (4 and 2 mm screen; Retsch Cutting Mill SM 200, Retsch, Haan, Germany). Ground hay, supplements (corn grain and beet pulp), orts, and fecal samples were then analyzed for DM (ref. [10]; method 930.15), OM (ref. [10]; method 942.05), NDF and ADF [11], and CP using the Kjeldahl procedure (Foss Analytics; Hillerød, Denmark; ref. [10]; method 976.05). For NDF analysis, sodium sulfite and alpha amylase were used. The method described by [12] was used for indigestible NDF analysis. Briefly, samples (0.6 g) were weighed into F57 bags (Ankom Technology; Macedon, NY, USA) that were then incubated for 288 h in the rumen of 2 cows. After incubation, the residues were analyzed for NDF as previously described.

In preparation for NH_3_-N analysis, ruminal fluid samples preserved with H_2_SO_4_ were first centrifuged (10,800× *g* for 20 min at 4 °C). The supernatant was then collected and analyzed using a phenol–hypochlorite assay [13]. In preparation for SCFA analysis, ruminal fluid samples preserved with H_2_PO_4_ were centrifuged (12,000× *g* for 10 min at 4 °C) before collection of the supernatant for additional centrifugation (16,000× *g* for 10 min at 4 °C). The supernatant from the second centrifugation step was then filtered (0.2 μm Nylon filter) and diluted (1:1) with distilled water. A gas chromatograph with a flame-ionization detector (GC-FID; 6890 Series, Hewlett-Packard; Palo Alto, CA, USA) was then used for SCFA analysis using the method described by [14].

Acidified urine composites were thawed at room temperature. Thereafter, samples were analyzed for total N using the Kjeldahl procedure (Foss Analytics; Hillerød, Denmark; ref. [10]; method 976.05). Urine creatinine, urine urea-N (UUN), and PUN concentrations were determined using commercial kits (Arbor Assays; Ann Arbor, MI, USA). A method adapted from [15] was used for urine allantoin and uric acid analysis. Briefly, quantification was carried out using an HPLC/MS (Waters Corporation, Milford, MA, USA) fitted with a reversed-phase column (C18, 5 µm particle size, 2 mm × 250 mm; Phenomenex, Torrance, CA, USA) using a 5% methanol mobile phase.

### 2.4. Calculations

The apparent total-tract digestibility of DM, OM, CP, NDF, and ADF was calculated as follows:

100 − 100 × ((iNDF concentration in feed/iNDF concentration in feces) × (nutrient concentration in feces ÷ nutrient concentration in feed)).

Urine output was estimated using the concentration of creatinine measured in urine, and BW and creatinine constant of 29 mg/kg BW per day [16] using the following equation:

Urine output, kg/d = (29 × BW^0.75^) ÷ creatinine concentration, mg/L

Apparent nitrogen balance was determined by difference (N intake − (fecal N + urine N excretion)).

To estimate the total absorption of purine derivatives (PDs), urine allantoin and uric acid excretion were used in the following equation:

PD_excreted_, mmol/d = 0.85(PD_absorbed_) + (0.385 BW^0.75^),

where 0.85 is the recovery of absorbed purines as PD and 0.385 BW^0.75^ is representative of purine excretion from endogenous sources. The flow of microbial N was calculated using the following equation:

Microbial N, g/d = 70(PD_absorbed_) ÷ (0.116 × 0.83 × 1000),

where 70 represents the N content of purines (mg N/mmol), 0.83 is the digestibility of those purines, and the ratio of purine-N:total N in rumen microbes is 11.6:100.

### 2.5. Statistical Analysis

All data were analyzed as a split-plot, Latin square design with teff hay harvest maturity as the whole plot and supplemental energy source as subplot. Fixed effects of period, harvest maturity, supplemental energy source, and harvest maturity × supplemental energy source interaction and a random effect of heifer (harvest maturity) were used in the MIXED procedure of SAS (version 9.4, SAS Inst. Inc., Cary, NC, USA). Repeated measures analysis of ruminal NH_3_-N data was carried out by including additional terms for time (hour) and fixed effect interactions with time in the previously described model. Modeling of variance–covariance structure of the repeated measures was conducted separately, and an appropriate structure was fitted using the lowest values of the fit statistics based on the Bayesian information criteria. Prior to analysis, residual distributions were evaluated for normality and homoscedasticity. Data are presented as least square means. Significance was declared at *p* < 0.05 and trends at 0.05 < *p* ≤ 0.10.

## 3. Results

### 3.1. Dietary Chemical Composition

Feedstuff chemical composition is presented in Table 1. Although statistical analysis was not conducted, the OM and CP content were both numerically greater for EH than LH teff hay. However, DM, ADF, and NDF content were comparable across the two stages of maturity. As expected, the CP content was greater, whereas the ADF and NDF content were lower, for corn grain compared to beet pulp.

### 3.2. Nutrient Intake and Apparent Total-Tract Digestibility

There was no harvest maturity × energy supplement interaction (*p* ≥ 0.80) for nutrient intake and apparent total-tract digestibility (Table 2). Although harvest maturity did not impact (*p* ≥ 0.11) DM, OM, and NDF intake, CP intake was greater (*p* < 0.01) for heifers fed EH than LH teff hay. Feeding supplemental beet pulp and corn grain resulted in an increase (*p* < 0.01) in DM and OM intake and a tendency for an increase (*p* = 0.06) in CP intake. However, ADF intake was greater (*p* < 0.01) and NDF intake tended to be greater (*p* = 0.07) for heifers fed supplemental beet pulp compared to CON heifers and heifers fed supplemental corn grain. Apparent total-tract DM, OM, NDF, ADF, and CP digestibility were greater (*p* ≤ 0.02) for heifers fed teff hay harvested at the EH than at the LH stage of maturity. Although there was no supplement effect (*p* ≥ 0.23) on apparent total-tract NDF, ADF, and CP digestibility, feeding supplemental beet pulp and corn grain resulted in a tendency for an increase in apparent total-tract DM (*p* = 0.09) and OM digestibility (*p* = 0.096).

### 3.3. Ruminal Fermentation Characteristics

There was no harvest maturity × energy supplement interaction (*p* ≥ 0.13) effect on ruminal pH and SCFA profile (Table 3). Harvest maturity of teff hay had no effect (*p* ≥ 0.17) on all measures of ruminal pH. However, feeding supplemental corn grain resulted in a decrease (*p* = 0.04) in mean ruminal pH compared to CON heifers and heifers fed beet pulp. Feeding supplemental corn grain also resulted in a tendency for a decrease (*p* = 0.09) in minimum ruminal pH compared to CON and beet pulp heifers. However, the maximum ruminal pH did not differ (*p* = 0.29) across treatments. The duration pH < 6.2 was greater (*p* = 0.049) for heifers fed supplemental grain compared to CON heifers and heifers fed supplemental beet pulp. The area under the curve for pH < 6.2 also tended to be greater (*p* = 0.08) for heifers fed supplemental corn grain compared to CON and beet pulp heifers. However, energy supplements had no effect (*p* ≥ 0.13) on both the duration and area under the curve for pH < 5.8. Although there was no dietary treatment effect (*p* ≥ 0.12) on the total SCFA concentration, the molar proportions of propionate, valerate, and isobutyrate, and the acetate:propionate ratio, the molar proportion of acetate was lower (*p* < 0.01) for heifers fed supplemental corn grain than CON heifers and heifers fed supplemental beet pulp. The molar proportion of butyrate, which was greater (*p* = 0.03) for heifers fed LH than EH hay, was also greater (*p* < 0.01) for heifers fed supplemental corn grain compared to CON heifers and heifers fed supplemental beet pulp. The molar proportion of isovalerate was greater (*p* < 0.01) for heifers fed supplemental corn grain compared to CON and beet pulp heifers. The molar proportion of total BCFA was lower (*p* < 0.01) for heifers fed supplemental beet pulp compared to CON and corn grain heifers.

### 3.4. Nitrogen Utilization

**Figure 1:**
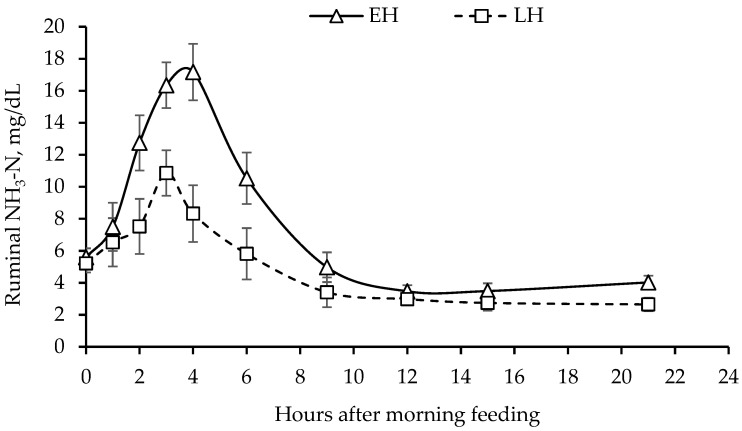
Ruminal NH_3_-N concentration for beef heifers fed either early-heading (EH) or late-heading (LH) teff hay and provided with no supplement (CON), supplemental beet pulp, or supplemental corn grain. Heifers were fed once daily at 0630 h. Harvest maturity, *p* = 0.01; energy supplement, *p* = 0.65; harvest maturity × energy supplement, *p* = 0.37; harvest maturity × time, *p* = 0.03; energy supplement × time, *p* = 0.89. The error bars reflect the SEM associated with time.

Except for urinary uric acid excretion, there was no harvest maturity × energy supplement interaction (*p* ≥ 0.26) for measures of N utilization (Table 4). Nitrogen intake, which was greater (*p* < 0.01) for EH than LH heifers, was also greater (*p* = 0.048) for heifers fed supplemental corn grain than CON heifers; however, it did not differ between heifers fed either supplemental corn grain or beet pulp. Although it tended to be greater (*p* = 0.07) for LH than EH heifers, fecal DM output did not differ (*p* = 0.62) across energy supplements. Fecal N excretion (% of N intake) was greater (*p* = 0.02) for EH than LH heifers; however, it did not differ (*p* = 0.77) across energy supplements. There was no dietary treatment effect (*p* ≥ 0.13) on total and fractional urine N and urea-N excretion, total N excretion, and apparent N retention. Urinary excretion of allantoin, uric acid, and total PD and estimated microbial N flow were greater (*p* < 0.01) for heifers fed EH than LH hay. However, there was no energy supplement effect (*p* ≥ 0.16) on urinary allantoin and total PD excretion and estimated microbial N flow. Ruminal NH_3_-N concentration tended to be greater (*p* = 0.095) for EH than LH heifers; however, there were no differences (*p* = 0.56) across energy supplement sources (Figure 1). Plasma urea-N concentration did not differ (*p* ≥ 0.11) across dietary treatments.

## 4. Discussion

Because both factors influence nutrient supply and animal performance, we evaluated the effects of feeding teff hay harvested at the EH or LH stage of maturity and providing either a non-structural (corn grain) or structural carbohydrate (beet pulp) as an energy supplement on nutrient intake, apparent total-tract nutrient digestibility, ruminal fermentation characteristics, and nitrogen utilization. There was no harvest maturity × energy supplement interaction for most measurements; therefore, the discussion primarily focuses on the main effects of harvest maturity of teff hay and supplemental energy source.

As expected, delaying the harvest of teff hay resulted in a decrease in CP content. However, OM, ADF, NDF, starch, and WSC content did not differ between EH and LH teff hay. Similar observations were made in other studies [3,5], with the lack of the anticipated increase in fiber accumulation with advancing maturity attributed to various factors including climatic and agronomic conditions that can potentially alter processes like lignification. Dry matter intake did not differ across harvest maturity in the present study. Together with the comparable OM, NDF, and ADF content between EH and LH hay, this explains the lack of a difference in intake of those nutrients across harvest maturity. However, as expected, heifers that consumed EH hay, which had a higher CP content, consumed more protein than heifers fed LH hay.

A key concern when feeding energy supplements is the potential decrease in forage intake, with the substitution rate influenced by several factors including supplementation level, supplement type, and supplementary daily meals [1,17]. In the current study, total diet intake was greater for heifers fed both supplements compared to CON heifers; however, the provision of either corn grain or beet pulp did not result in a decrease in forage intake. After reviewing numerous studies in grazing cattle, ref. [18] concluded that a supplementation rate of <0.5% of BW was unlikely to cause a substantial decrease in forage intake. Similarly, after using the meta-analytical approach to evaluate data collected from studies where nonlactating cattle were fed forage for ad libitum intake, ref. [19] observed that substitution effects typically occur when a high level of supplementation (>0.7% of BW) is used and when forage intake alone is >1.75% of BW. In the present study, the supplementation level was 0.5% of BW; therefore, this could potentially account for the lack of a decrease in forage intake. Moreover, the intake of forage, which was not restricted, was 1.15% of BW (SD = 0.144) in this study.

The amount of energy supplement that could lead to compromised forage intake when fed has been suggested to be lower for NSCs compared to structural carbohydrates [20]. This is because the potential depression in ruminal pH, which compromises fiber digestibility, slows down the digesta passage rate, and, in turn, reduces DMI, is expected to be greater for NSCs that have a faster rate and extent of ruminal fermentation compared to structural carbohydrates. Therefore, a supplementation rate as low as 0.25% of BW for grain has been reported to reduce forage utilization [20]. In the present study, feeding supplemental corn grain at 0.5% of DM resulted in a decrease in mean and minimum ruminal pH and an increase in the duration and area with pH < 6.2 compared to the CON diet. However, across treatments, there was no difference in duration and area with pH < 5.8, the threshold below which ruminal fiber digestion is compromised [21]. Although ruminal fiber digestion was not measured in our study, there was no supplement effect on apparent total-tract fiber digestion. Therefore, it is possible that the observed decrease in ruminal pH for heifers fed corn grain did not result in a substantial decrease in fiber digestion in the rumen and, consequently, forage intake.

Increasing the concentrate feeding frequency per day has also been reported to lead to an increase in the forage substitution rate [22]. This could be related to ruminal pH being depressed to a greater extent and for a longer duration, thereby reducing the growth and activity of fibrolytic microbes. In the present study, supplements, which were offered once per day, were consumed within the first hour of being fed. Therefore, this could possibly explain the lack of a diet effect on forage intake, including for heifers fed corn grain. In another study, ref. [23] also did not observe a decrease in forage intake and diet digestibility when corn grain was supplemented at < 0.6% of BW in beef steers grazing on ryegrass. Unlike in our study and the study by [23], ref. [24] reported a decrease in DMI and fiber digestibility after providing supplemental corn grain compared to beet pulp to lambs fed low-quality hay. It is important to note that the supplement inclusion level was higher (at least 1.75% of BW) than in the present study. However, even at a high dietary inclusion level, beet pulp did not compromise either intake or digestibility in the study by [24], highlighting the greater appeal of using fibrous byproducts that are highly digestible compared to starchy feeds like corn grain in supplementation programs.

As a result of the lack of a harvest maturity effect on DMI, and comparable carbohydrate profile, it was not surprising that ruminal total SCFA production and all pH variables did not differ between EH and LH heifers in the present study. Feeding supplemental corn grain resulted in a decrease in the ruminal molar proportion of acetate and an increase in the ruminal molar proportion of butyrate compared to the control and beet pulp diets. This possibly reflects a decrease in dietary fiber and an increase in the dietary starch supply for heifers fed corn grain [25]. Similarly, the increase in the molar proportion of isovalerate and total BCFA for heifers fed corn grain suggests a greater increase in ruminal CP and, possibly, branched-chain amino acid supply, compared to heifers fed supplemental beet pulp [26]. Although ruminal and post-ruminal nutrient digestion were not measured in the current study, apparent total-tract DM and OM digestibility tended to be greater for heifers fed supplemental corn grain and beet pulp compared to CON heifers. This possibly occurred because of the increase in consumption of those nutrients. However, feeding corn grain did not lead to a decrease in apparent total-tract fiber digestibility, which can occur due to a decrease in ruminal pH suppressing the growth and activity of cellulolytic microbes and, thus, ruminal fiber digestion. However, it is important to note that post-ruminal digestion of fiber can also potentially compensate for a decrease in ruminal digestion [27]. There are indications that outcomes when feeding supplemental energy sources could also be impacted by ruminal degradable protein (RDP) supply. For instance, ref. [28] observed that apparent total-tract NDF digestibility, which was compromised when the RDP supply was insufficient, was increased when the RDP supply was close to sufficient. Although not measured in the present study, the lower ruminal NH_3_-N concentration for LH heifers suggests a lower RDP supply than for EH heifers. However, we did not observe a maturity × fermentable CHO supply interaction for apparent total-tract NDF digestibility. Apparent total-tract digestibility of DM, OM, fiber, and CP digestibility was greater for heifers fed EH than LH teff hay. Others [3,5] also reported greater apparent total-tract DM and CP digestibility for EH than LH hay, which potentially increases nutrient supply.

A ruminally fermentable energy supply is a key determinant of microbial protein synthesis [29,30]. Therefore, we anticipated an increase in microbial protein supply with energy supplements, especially corn grain. However, that was not the case, as there was no supplement effect on urinary purine derivative excretion and, thus, estimated microbial N flow. An increase in fermentable energy supply increases the capture of N sources including NH_3_-N for microbial protein synthesis [30,31]. Therefore, the lack of a supplement effect on ruminal NH_3_-N concentration could be indicative of the additional energy from supplements potentially not being substantial enough to increase microbial growth. Since low ruminal pH can compromise microbial growth, the lower minimum pH for heifers fed supplemental corn grain could have also prevented the anticipated increase in microbial N flow. Unlike the present study, ref. [32] reported a decrease in the ruminal NH_3_-N concentration and pool size and increased ruminal outflow of overall microbial N and microbial N synthesized from NH_3_-N following the intraruminal dosing of pure corn starch. The high dosage rate (20% of dietary DMI) in that study possibly led to a consequential increase in readily fermentable energy supply that impacted ruminal N metabolism. This was despite a noted decrease in ruminal pH, especially 2 to 4 h post-dosing.

In addition to the fermentable energy supply, the other major determinant of microbial protein synthesis is ruminal N supply from peptides, amino acids, and NH_3_-N. Of the 3 N sources, only NH_3_-N, which supplies up to 80% of the N required for microbial growth [33], was quantified in this study. There was no supplement effect on ruminal NH_3_-N concentration over time, possibly in part because the increase in N intake was marginal. However, N intake was greater for EH than LH heifers, reflecting the greater CP content of EH compared to LH hay, since DMI did not differ across harvest maturity. Although ruminal CP digestibility was not measured in the present study, it probably was also greater for EH than LH hay since fecal N excretion (% of N intake) was lower for EH than LH heifers. This is supported by the higher ruminal NH_3_-N concentration for EH than LH heifers. A ruminal NH_3_-N concentration of 5.0 to 11.8 mg/dL has been suggested as the range needed to maximize microbial growth depending on factors such as dietary energy supply [30,34]. Since the duration of the ruminal NH_3_-N concentrations above the 5.0 or 11.8 mg/dL thresholds was longer for EH than LH heifers, this could account for the greater urinary excretion of PD and estimated microbial N flow. However, the greater microbial N supply did not translate to better growth performance, as reflected by the lack of a difference in apparent N retention, which was negative, suggesting that the metabolizable protein supply was inadequate across harvest maturity. Although not measured, it is possible that the RDP supply was also deficient across harvest maturity. Since the RDP supply can potentially influence the effectiveness of energy supplementation [28], an RDP deficiency across harvest maturity could explain the lack of the anticipated harvest maturity × energy supplement interaction for all measurements in the present study. Therefore, this necessitates additional research focused on both energy and protein supplementation for cattle fed teff hay harvested at different stages of maturity.

## 5. Conclusions

There was no harvest maturity × energy supplement interaction for all measurements. However, feeding supplemental corn led to a decrease in mean pH and an increase in the duration of pH < 6.2; however, this did not compromise apparent total-tract nutrient digestibility. Although feeding both supplements resulted in an increase in DM and OM intake, it had no impact on ruminal N metabolism and microbial N flow. On the other hand, although N intake, apparent total-tract DM, OM, fiber and CP digestibility, ruminal NH3-N concentration, and estimated microbial N flow were all greater for EH than LH heifers, this did not lead to an increase in N retention. The negative N retention across harvest maturity suggests the need for both energy and protein supplements to improve growth performance when feeding teff hay to beef cattle.

## Figures and Tables

**Table 1:** Chemical composition of feedstuff (dry matter basis, %).

Item	Feedstuff ^1^			
	EH Teff Hay	LH Teff Hay	Corn Grain	Beet Pulp
Chemical analysis				
Dry matter	92.5 ± 0.46	92.7 ± 0.30	86.5 ± 1.60	91.8 ± 0.20
Organic matter	89.3 ± 0.58	86.7 ± 1.32	98.7 ± 0.17	86.0 ± 0.43
Crude protein	14.1 ± 0.22	12.4 ± 0.32	8.6 ± 0.37	6.9 ± 0.09
Acid detergent fiber	30.7 ± 0.67	31.6 ± 0.01	1.6 ± 0.06	30.7 ± 0.25
Neutral detergent fiber	62.9 ± 0.87	63.4 ± 1.28	9.9 ± 0.58	45.3 ± 0.49
Starch	0.06 ± 0.014	1.05 ± 0.071	73.3 ± 5.8	0.90 ± 0.07
Water soluble carbohydrates	4.60 ± 0.141	3.45 ± 0.071	-	-

^1^ EH = early-heading, LH = late-heading.

**Table 2:** Nutrient intake and apparent total-tract nutrient digestibility for beef heifers fed either early-heading (EH) or late-heading (LH) teff hay and provided with no supplement (CON), supplemental beet pulp, or supplemental corn grain.

Variable	Harvest Maturity		SEM	Supplement			SEM	*p*-Value ^1^		
	EH	LH		CON	Beet Pulp	Corn Grain		HM	SP	HM × SP
Intake ^2^, kg/d										
DM	12.2	11.3	0.37	9.8 ^a^	12.6 ^b^	12.8 ^b^	0.47	0.13	<0.01	0.90
OM	10.7	10.2	0.36	8.6 ^a^	11.0 ^b^	11.6 ^b^	0.44	0.34	<0.01	0.90
NDF	7.37	6.73	0.255	6.70	7.73	6.72	0.312	0.11	0.07	0.89
ADF	3.73	3.35	0.124	3.31 ^a^	4.12 ^b^	3.20 ^a^	0.151	0.055	<0.01	0.91
CP	1.56 ^x^	1.32 ^y^	0.050	1.32	1.45	1.55	0.059	<0.01	0.06	0.90
ATTD ^3^, %										
DM	55.2 ^x^	43.1 ^y^	2.61	42.6	52.8	52.1	3.20	<0.01	0.09	0.80
OM	59.0 ^x^	48.8 ^y^	2.40	48.1	56.7	57.0	2.94	0.01	0.096	0.84
NDF	60.2 ^x^	48.8 ^y^	2.25	54.6	56.5	52.4	2.75	<0.01	0.58	0.88
ADF	57.4 ^x^	47.3 ^y^	2.55	51.1	57.0	48.9	3.12	0.02	0.23	0.91
CP	58.7 ^x^	44.2 ^y^	3.10	52.0	53.4	49.1	3.80	<0.01	0.72	0.93

^a,b^ Differing letters within harvest maturity indicate a difference (*p* ≤ 0.05). ^x,y^ Differing letters within supplemental energy source indicate a difference (*p* ≤ 0.05). ^1^ HM = harvest maturity, SP = energy supplement, and HM × SP = harvest maturity × energy supplement; ^2^ DM = dry matter, OM = organic matter, NDF = neutral detergent fiber, ADF = acid detergent fiber, CP = crude protein; ^3^ ATTD = apparent total-tract digestibility.

**Table 3:** Ruminal fermentation characteristics for beef heifers fed either early-heading (EH) or late-heading (LH) teff hay with no supplement (CON) or supplemental beet pulp or corn grain.

Variable	Harvest Maturity		SEM	Supplement			SEM	*p*-Value ^1^		
	EH	LH		CON	Beet Pulp	Corn Grain		HM	SP	HM × SP
Intake, kg/d										
Total	12.2	11.3	0.37	9.8 ^a^	12.6 ^b^	12.8 ^b^	0.47	0.13	<0.01	0.90
Forage	9.83	8.88	0.348	9.83	8.88	9.35	0.427	0.08	0.33	0.85
pH										
Mean	6.38	6.37	0.048	6.46 ^a^	6.41 ^a^	6.25 ^b^	0.046	0.79	0.04	0.60
Minimum	5.91	5.96	0.088	6.06	6.02	5.73	0.090	0.74	0.09	0.53
Maximum	6.75	6.78	0.036	6.83	6.75	6.72	0.044	0.55	0.29	0.50
DUR ^2^										
<6.2	319	304	93.7	146 ^a^	239 ^a^	551 ^b^	93.8	0.92	0.049	0.36
<5.8	30.4	82.9	28.71	8.1	36.7	125.2	40.13	0.25	0.13	0.14
AUC ^3^										
<6.2	57.2	89.1	28.48	23.1	50.0	146.6	33.30	0.43	0.08	0.18
<5.8	3.59	16.03	6.176	0.46	6.10	22.87	7.783	0.17	0.15	0.13
Acidosis index ^4^	0.275	1.321	0.5113	0.045	0.483	1.867	0.6518	0.17	0.15	0.14
SCFA ^5^										
Total, mmol/L	80.1	75.7	2.15	77.3	77.9	78.4	3.49	0.22	0.95	0.74
Acetate, %	77.6	77.0	0.63	78.9 ^a^	78.2 ^a^	74.9 ^b^	0.65	0.54	<0.01	0.71
Propionate, %	15.4	14.3	0.47	14.5	15.0	15.2	0.58	0.12	0.67	0.97
Butyrate, %	5.27 ^x^	6.83 ^y^	0.317	4.84 ^a^	5.29 ^a^	8.01 ^b^	0.334	0.03	<0.01	0.44
Valerate, %	0.412	0.475	0.0379	0.455	0.389	0.488	0.0464	0.27	0.35	0.72
Isobutyrate, %	0.662	0.687	0.0371	0.674	0.624	0.724	0.0350	0.66	0.12	0.79
Isovalerate, %	0.584	0.655	0.0361	0.616 ^a^	0.510 ^a^	0.733 ^b^	0.0367	0.24	<0.01	0.53
Total BCFA ^6^, %	1.24	1.34	0.073	1.29 ^a^	1.13 ^b^	1.46 ^a^	0.064	0.40	<0.01	0.51
A:P ratio ^7^	5.04	5.42	0.184	5.48	5.22	5.01	0.225	0.18	0.37	0.96

^a,b^ Differing letters within harvest maturity indicate a difference (*p* ≤ 0.05). ^x,y^ Differing letters within supplemental energy source indicate a difference (*p* ≤ 0.05). ^1^ HM = harvest maturity, SP = energy supplement, and HM × SP = harvest maturity × energy supplement; ^2^ DUR = duration of pH, min/d; ^3^ AUC = area under curve, pH × min/d; ^4^ area under curve pH < 5.8/kg DMI; ^5^ short-chain fatty acid; ^6^ branched-chain fatty acid; ^7^ acetate:propionate ratio.

**Table 4:** Measures of nitrogen (N) utilization for beef heifers fed either early-heading (EH) or late-heading (LH) teff hay and provided with no supplement (CON), supplemental beet pulp, or supplemental corn grain.

Variable	Harvest Maturity		SEM	Supplement			SEM	*p*-Value ^1^		
	EH	LH		CON	Beet Pulp	Corn Grain		HM	SP	HM × SP
Intake										
N, g/d	249 ^x^	212 ^y^	8.8	211 ^a^	232 ^ab^	249 ^b^	11.1	<0.01	0.048	0.90
Fecal excretion										
DM, kg/d	5.42	6.30	0.315	5.58	5.88	6.12	0.386	0.07	0.62	0.92
N, g/d	103	117	8.5	99	106	126	9.6	0.30	0.08	0.96
N, % of N intake	41.3 ^x^	55.6 ^y^	3.84	48.0	46.6	50.7	4.87	0.02	0.77	0.94
Urinary excretion										
Total output, kg/d	17.1	12.8	1.75	16.5	15.1	13.3	2.22	0.095	0.58	0.82
N, g/d	178	145	17.3	179	165	139	22.0	0.19	0.42	0.50
Urea-N, g/d	118	91	12.5	116	108	90	15.8	0.13	0.47	0.57
Urea-N, % urine N	66.7	62.0	2.23	64.6	64.2	64.3	2.83	0.15	0.99	0.93
Total N, % N intake	71.8	70.9	8.76	86.0	71.7	56.3	11.11	0.94	0.18	0.51
Allantoin, mmol/d	199 ^x^	119 ^y^	17.2	167	154	156	21.9	<0.01	0.90	0.50
Uric acid, mmol/d	55.0 ^x^	22.8 ^y^	4.18	36.6	32.8	47.3	5.30	<0.01	0.16	0.051
Total PD ^2^, mmol/dL	254 ^x^	142 ^y^	18.8	203	187	210	23.9	<0.01	0.83	0.26
Microbial N, g/d	117.2 ^x^	50.4 ^y^	11.40	86.7	77.2	87.5	13.3	<0.01	0.83	0.29
Total N excretion,										
g/d	281	261	20.3	278	271	264	25.7	0.49	0.92	0.54
% N of intake	113	126	11.0	134	118	107	12.4	0.39	0.37	0.58
N retention, g/d	−31.9	−48.9	22.96	−67.2	−38.9	−14.9	29.1	0.60	0.43	0.67
Rumen NH_3_-N, mg/dL	10.04	7.98	0.816	9.21	9.67	8.16	1.000	0.095	0.56	0.87
Plasma urea-N, mg/dL	13.6	13.5	0.04	13.5	13.7	13.5	0.05	0.22	0.11	0.27

^a,b^ Differing letters within harvest maturity indicate a difference (*p* ≤ 0.05). ^x,y^ Differing letters within supplemental energy source indicate a difference (*p* ≤ 0.05). ^1^ HM = harvest maturity, SP = energy supplement, and HM × SP = harvest maturity × energy supplement; ^2^ PD = purine derivatives.

## Data Availability

The data presented in this study are available on request from the corresponding author. The data are not publicly available as it also forms part of an ongoing study.
